# Screening of ‘Y’ chromosome microdeletions in Iranian infertile males

**DOI:** 10.4103/0974-1208.38973

**Published:** 2008

**Authors:** Ali Mohammad Malekasgar, Hayat Mombaini

**Affiliations:** Department of Biochemistry, Genetic Unit, Ahwaz Jondishapur University of Medical Sciences, Iran

**Keywords:** Azoospermia, infertility, oligospermia, Y-chromosome microdeletions

## Abstract

**BACKGROUND:**

It has been hypothesized that microdeletions of Yq may account for a significant proportion of men with infertility. Three nonoverlapping regions, referred to as “azoospermia factors” (AZFa, b, c from proximal to distal Yq) have been defined as spermatogenesis loci and deletions in these regions have been shown to be pathogenically involved in male infertility associated with azoospermia or severe oligospermia.

**AIMS:**

Evaluation the frequency of Y chromosome microdeletions in Iranian population.

**MATERIALS AND METHODS:**

Fifty infertile men were selected. Semen analysis was done and on the basis of the mean sperm count, all patients were categorized into azoospermia and oligozoospermia, groups. Blood samples were obtained for DNA extraction and chromosomal analysis. Genomic DNA was extracted from blood lymphocytes and amplified by sequence tagged sites-polymerase chain reaction (STS-PCR) method to determine the presence of microdeletions in AZF locus. A total of 34 STS primers including two controls were selected to identify microdeletions of Y chromosome on each subject.

**RESULTS AND CONCLUSION:**

26/50 cases (52%) showed deletion of at least one of the STS Marker. Totally 41 microdeletions was observed. A total of 17 cases (34%) had deletion in one STS. Four oligospermia cases (8%) had deletion in 2 STS site. Three azoospermia cases (6%) had again deletion in 2 STS site, but in different STSs. One case had three deletions in three STS site and finally one individual had seven deletions in AZF locus. The overall frequency of Y chromosome microdeletions observed in the present study was found to be 26/50 (52%). Comparison of our data with the result of other investigators world wide shows that the incidence of Yq microdeletions in Iranian population is much higher than international frequency. Our data agree with other studies regarding microdeletions of AZFc, but for microdeletions of AZFa (14.6%) our results is much higher and differ significantly with many studies.

Nowadays, infertility study is important, because of having access to advanced reproductive technologies. Techniques of ovulation stimulating, *in vitro* fertilization (IVF), and microinjection or intracytoplasmic sperm injection (ICSI) are promising treating methods for infertile couples. There is evidence that 60% cases of male infertility have an underlying genetic basis.[1] Y chromosome microdeletions are small deletions in the distal euchromatic region of the long arm of the Y chromosome, in intervals 5 and 6.[2] This region mainly comprises the azoospermia factors AZF a, b, d, and c. Genes like deleted in azoospermia (DAZ) and RNA binding motif (RBM) have proved to be of particular interest in this region and occur in multiple copies.[3] Search for Y chromosome microdeletions is essentially focused on screening the azoospermia factor (AZF) region through sequence tagged sites (STSs) spanning this area. Early studies attempted to assign specific infertility phenotypes to each region. However, Pryor *et al.*[4] showed that men with mild oligozoospermia and those with normal sperm counts, but abnormal sperm morphology can have microdeletions in AZFa, AZFb, or AZFc loci.

Concerning fertility, not only physical and physiological factors are important, but mental and emotional parameters have rules too.[5]

There is no standard definition for “infertility,” yet conventionally, Eliasson,[6] definition for infertility is being used. The world “infertility” is used after 1 year of efforts to reproduce, without using any contraceptive methods, yet no pregnancy has been occurred and we call it “sterility” when there is not any chance for reproduction.[6] Infertility accounts for 15% of all couples and about half of these cases belong to males. Unexpectedly, infertile males show structural disorders in spermatozoids that disable the sperm movement.[7]

There may be many factors in male fertility including, anatomical, hormonal, infectious, immunologic, mental, environmental, idiopathic, and genetical.

Genetic factors in male infertility include monogenic and chromosomal, such as cystic fibrosis, klinefelter, and translocations. One of the main genetic factors in male fertility is microdeletions of AZF, s locus in Y chromosome [Figure 1].

**Figure 1:**
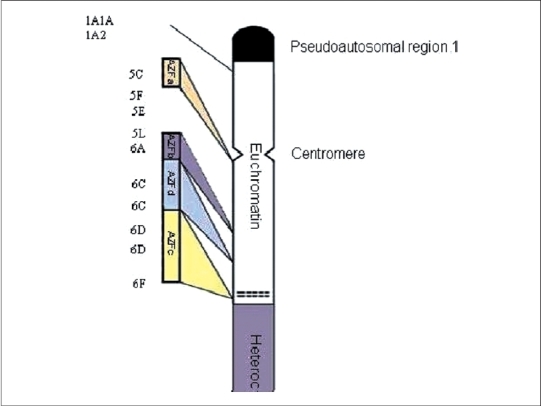
Y chromosome

In 1986, Vergnaud[2] draw a molecular map for Y chromosome and he divided this chromosome into seven intervals according to deletions he found in infertile men. Most of the deletions in infertile men he found were located in interval VI. Based on these observations, he defined an area in q arm of Y chromosome that was responsible for controlling spermatogenesis. This area contains AZF locus which is located in Yq 11.23 and have five million base pair.[2,8]

It seems that AZFa expression is related to proliferation of spermatogonia or their differentiation before and during the maturation period whereas AZFb expression is confined to pre-meiosis stage and spermatocytal stage. Function of genes in AZFC and specially AZFd is not well known.[9,10] Some of the chromosomal abnormalities can disturb germinal cell maturation especially in males. The rate of chromosomal abnormalities in infertile males is reported to be 7-13%.[11–13]

There is paucity of data on Y chromosome microdeletions in the Iranian population. The present study was undertaken to determine the frequency and find common loci of Y chromosome microdeletions among infertile men in Iran. We also try to evaluate any variation in the incidence of Y chromosome microdeletions in Iranian infertile males as compared to other countries.

## MATERIALS AND METHODS

### Patient selection

Fifty infertile males with a history of male factor infertility were selected from couples attending Mehr Infertility Institute at Rasht city of Iran for treatment. Every patient was asked to provide a minimum of 1 mL semen specimens, after sexual abstinence of at least 3 days. The specimens were evaluated on the basis of the criteria of the World Health Organization.[14] Semen analysis was performed for sperm-count, motility, viability, and morphology. The patients were then categorized into two main groups according to semen analyses. One group of azoospermia (AZ), consisting of 31 patients and the second group with oligozoospermia (sperm count below 20 million per mL), consisting of 19 patients. In addition, blood samples were obtained for DNA extraction and chromosomal analysis.

### Selected STS primers for screening Y-linked sequence

Genomic DNA was extracted from peripheral venous blood lymphocytes using a standard phenol-chloroform protocol. Screening for Yq microdeletions was carried out in patients, using polymerase chain reaction (PCR) techniques by amplifying 34 different sequence tagged sites (STSs) corresponding to different azoospermia factors (AZFs) loci [Table 1], these include:

**Table 1:** Primer characteristics used in this study

Sr. number	Product length	Primer name	Locus	Primer sequence
1	220 bp	AZFa	AZFa (prox-2)	5-GGTTCCTGAACAGGGGACT-3
				5-GGCAGCAGAAGGGCCTCTC-3
2	209 bp	Y81	AZFa start	5-AGGCACTGGTCAGAATGAAG-3
				5-AATGGAAAATACAGCTCCCC-3
3	495 bp	ZFY	Control	5′-ACCRCTGTACTGACTGTGATTACAC -3′
				5′-GCACYTCTTTGGTATCYGAGAAAGT -3′
4	320 bp	sY86	AZFa	5-GTGACACACAGACTATGCTTC-3′
				5′-ACACACAGAGGGACAACCCT-3
5	274 bp	sY127	AZFb	5′-GGCTCACAAACGAAAAGAAA-3′
				5′-CTGCAGGCAGTAATAAGGGA-3′
6	350 bp	sY254	AZFc	5′-GGGTGTTACCAGAAGGCAAA-3′
				5′-GAACCGTATCTACCAAAGCAGC-3′
7	326 bp	sY84	AZFa	5′-AGAAGGGTCTGAAAGCAGGT-3′
				5′-GCCTACTACCTGGAGGCTTC-3′
8	301 bp	sY134	AZFb	5′-GTCTGCCTCACCATAAAACG-3′
				5′-ACCACTGCCAAAACTTTCAA-3′
9	126 bp	sY255	AZFc	5′-GTTACAGGATTCGGCGTGAT-3′
				5′-CTCGTCATGTGCAGCCAC-3′
10	249 bp	USP9Y	AZFa	5′-CTTCACACAAATGCGTTTCA-3′
				5′-TGCAATTATTTGAACAAACATGA-3′
11	311 bp	Y143	AZFb	5′-GCAGGATGAGAAGCAGGTAG-3′
				5′-CCGTGTGCTGGAGACTAATC-3′
12	139 bp	Y153	AZFd	5′-GCATCCTCATTTTATGTCCA-3′
				5′-CAACCCAAAAGCACTGAGTA-3′
13	285 bp	Y157	AZFc	5′-CTTAGGAAAAAGTGAAGCCG-3′
				5′-CCTGCTGTCAGCAAGATACA-3′
14	173 bp	Y130	AZFa	5′-AGAGAGTTTTCTAACAGGGCG-3′
				5′-TGGGAATCACTTTTGCAACT-3′
15	177 bp	Y133	AZFb	5′-ATTTCTCTGCCCTTCACCAG-3′
				5′-TGATGATTGCCTAAAGGGAA-3′
16	190 bp	Y121	AZFa/b	5′-AGTTCACAGAATGGAGCCTG-3′
				5′-CCTGTGACTCCAGTTTGGTC-3′
17	109 bp	Y124	AZFb	5′-CAGGCAGGACAGCTTAAAAG-3′
				5′-ACTGTGGCAAAGTTGCTTTC-3′
18	228 bp	Y128	AZFb	5′-GGATGAGACATTTTTGTGGG-3′
				5′-AGCCCAATGTAAACTGGACA-3′
19	125 bp	Y152	AZFd	5′-AAGACAGTCTGCCATGTTTCA-3′
				5′-ACAGGAGGGTACTTAGCAGT-3′
20	472 bp	SRY (Y14)	Control	5′-GAATATTCCCGCTCTCCGGA-3′
				5′-GCTGGTGCTCCATTCTTGAG-3′
21	Variable	Y87	AZFa	5′-TCTGTTGCTTGAAAAGAGGG-3′
				5′-ACTGCAGGAAGAATCAGCTG-3′
22	Variable	Y90	AZFa	5′-CAGTGCCCCATAACACTTTC-3′
				5′-ATGGTAATACAGCAGCTCGC-3′
23	Variable	Y109	AZFb	5′-AGGAGATGTCAGGACTATCAGC-3′
				5′-TCCATCCAGCTGGTCATATT-3′
24	Variable	Y117	AZFb	5′-GTTGGTTCCATGCTCCATAC-3′
				5′-CAGGGAGAGAGCCTTTTACC-3′
25	Variable	Y155	AZFc	5′-ATTTTGCCTTGCATTGCTAG-3′
				5′-TTTTTAAGCCTGTGACCTGG-3′
26	Variable	Y146	AZFc	5′-ACAAAAATGTGGCTCAGGGA-3′
				5′-AAATAGTGTGCCCACCCAAA-3′
27	Variable	Y182	AZFa	5′-TCAGAAGTGAAACCCTGTATG-3′
				5′-GCATGTGACTCAAAGTATAAGC-3′
28	Variable	Y157	AZFc	5′-CTTAGGAAAAAGTGAAGCCG-3′
				5′-CCTGCTGTCAGCAAGATACA-3′
29	Variable	Y158	AZFc	5′-CTCAGAAGTCCTCCTAATAGTTCC-3′
				5′-ACAGTGGTTTGTAGCGGGTA-3′
30	Variable	Y276	AZFc	5′-CCTACCGCATCAGTGAATTTC-3′
				5′-TCTGTATGTGGAGTACACATGG-3′
31	Variable	Y283	AZFc	5′-CAGTGATACACTCGGACTTGTGTA-3′
				5′-GTTATTTGAAAAGCTACACGGG-3′
32	Variable	Y238	AZFc	5′-AACAAGTGAGTTCCACAGGG-3′
				5′-GCAAAGCAGCATTCAAAACA-3′
33	Variable	Y277	AZFc	5′-GGGTTTTGCCTGCATACGTAATTA-3′
				5′-CCTAAAAGCAATTCTAAACCTCCAG-3′
34	Variable	Y272	AZFc	5′-GGTGAGTCAAATTAGTCAATGTCC-3′
				5′-CCTTACCACAGGACAGAGGG-3′

For AZFa region: sY121, USP9G, sY182, sY90, sY87, sY86, sY84, sY81, and AZFa

For AZFb region: sY134, sY133, sY130, sY128, sY127, sY124, sY117, sY109, and sY114

For AZFc region: sY272, sY255, sY254, sY151, sY158, sY157, sY155, sY146, sY283, sY277, sY238, and sY276

For AZFd region: sY152, and sY153

Control markers SRY and ZFY

Besides using the external control marker, two other markers “internal control markers” were applied as well. For microdeletion detection in Yq chromosome, one of the most proper internal controls is ZFY marker because this primer can profile a unique segment in males, so negative control (female) lacks this and dose not reveals any band on agarose gel. Another internal control is SRY primer. This primer is applied to prove the presence of specific sequence [testicular determining factor (TDF)] in short arm of Y chromosome especially when ZFY gene is absent, like XX male.

A total of 50-100 ng of genomic DNA was used as template in 25 µL reaction mix, 1× amplification buffer, 1 mmol dNTPs, 10-25 pmol of each primer and 1.25 IU of *Taq* DNA polymerase. After an initial denaturation step of 5 min, each PCR reaction was carried at the annealing temperature specific for each primer pair. The PCR products were separated on 2-3% agarose gels stained with ethidium bromide, on the basis of the size of the product obtained. In case of any failure in amplification in of samples, two additional PCRs were performed to confirm the absence of the unamplified STSs.

### Conformation of the observed microdeletions by multiplex PCR

To conform the accuracy of single PCR procedures for observed microdeletions in first step, a multiplex PCR was performed to primers; sY277, sY182, sY283, sY158, and sY153. Sample of patients who had microdeletions in first step, along with related primers and one of the controls were used for multiplex PCR.

### Cytogenetic analysis

Chromosomal analysis was performed on phytohemagglutinin (PHA)-stimulated peripheral lymphocyte cultures using standard cytogenetic methods. Metaphase chromosomes of 21 patients with varicocele and unexplained infertility were analyzed using Giemsa-Trypsin-Giemsa (GTG) banding. A total of 15-20 metaphases were analyzed per individual and, in cases of suspected mosaicism; the numbers of metaphases were increased to a total of 40 for analysis. A resolution of 400-band stage was considered as a minimum.

## RESULTS AND DISCUSSION

On the basis of semen analysis 31/50 (63.4%) of the individuals were azoospermic and 19/50 (36.6%) were sever oligozoospermic. Clinical findings on presentation are shown in Table 2.

**Table 2:** Patient's clinical data and some associated diseases

Patient’s	Age	Spermogram	Associated diseases					Others
				
no.			Varicocele	Diabetes	Parotitis	Impotence	Family history
1	43	Oligozoospermia			†			
2	30	Azoospermia			†			
3	31	Oligozoospermia						
4	38	Oligozoospermia			*			
5	46	Oligozoospermia			†			
6	37	Azoospermia			*		*	
7	27	Azoospermia			*			
8	24	Azoospermia					*	
9	28	Azoospermia			*		*	
10	27	Azoospermia						
11	35	Oligozoospermia	+		*			
12	35	Azoospermia			†		*	
13	39	Azoospermia			*		*	
14	36	Azoospermia						Orchitis with sever testicular atrophy
15	27	Azoospermia						
16	24	Oligozoospermia			*			
17	45	Azoospermia			*	*		Start with oligo-changed to azoo
18	26	Azoospermia			*			
19	31	Azoospermia						Sertoli cell syndrom
20	34	Oligozoospermia						
21	27	Oligozoospermia			†			
22	38	Azoospermia						Sever spermatocytic arrest
23	33	Azoospermia						Germ cell aplasia & Sertoli cell syndrom
24	28	Azoospermia						
25	33	Oligozoospermia						
26	35	Azoospermia						
27	40	Oligozoospermia				*		
28	35	Azoospermia			†		*	Germ cell aplasia & Sertoli cell syndrom

* Positive

† Uncertain

The chromosomal abnormalities involving structural autosomal aberrations were not detected. Only two anomalies were detected in idiopathic infertile individuals, one with 47, XXY and one with marker chromosome. The origin of marker chromosome could not be evaluated. No significant differences were detected in the hormone profiles of azoospermic or severely oligozoospermic patients with or without abnormal karyotype.

## Y chromosome microdeletions

Y chromosome microdeletions were present in 26/50 (52%) of the individuals. Sixteen out of 26 of observed microdeletions were among azoospermic group, witch comprises 27/41 of total microdeletions [Tables 3a and b]. Out of 16 azoospermic cases with microdeletions, one patient (no. 34) had microdeletions in seven site (sY254, sY277, sY283, sY153, sY145, sY255, and sY157), another azoosperm patient (No. 30) had microdeletions in three site (sY157, sY238, and sY254), three azoosperm patients (6, 23, and 49) each had microdeletions in two different site and eleven patients had each microdeletion in one site. Ten out of 26 of patients with microdeletions were among sever oligozoospermic males witch comprises 14/41 of total microdeletions observed. Again out of these 10 severs oligozoospermic cases with microdeletions, four patients (no. 2, 5, 11, and 40) each had microdeletions in two different sites and six patients had each microdeletion in one site.

**Table 3a:** Diagram showing the pattern of deletions in infertile men

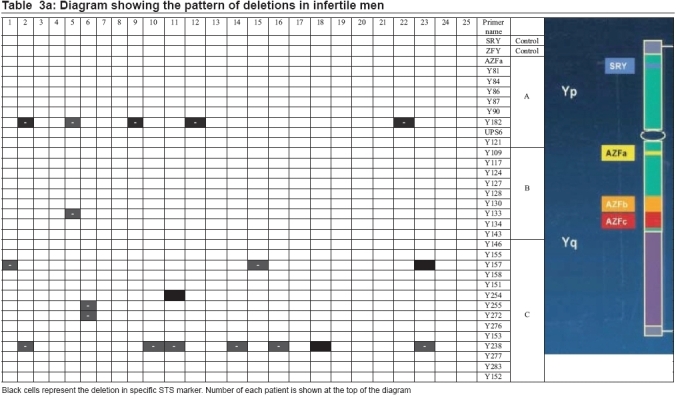

**Table 3b:** Diagram showing the pattern of deletions in infertile men

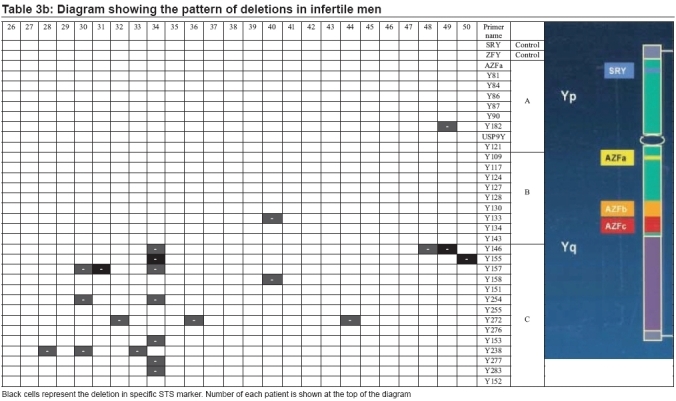

**Figure 2:**
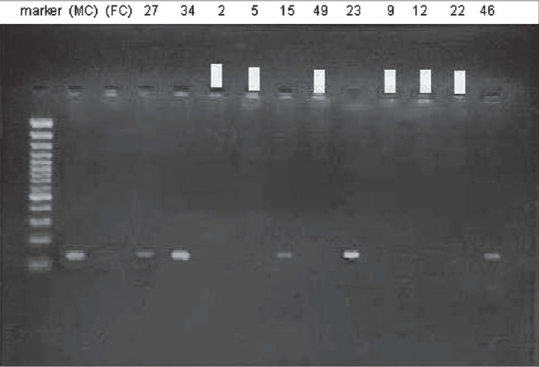
Electroforesis results of PCR product amplified by sY182 primer in some patients. Patient's number is indicated at top. Arrows represent the deleted subjects. MC, male control; FC, female control

Of 41 microdeletions found in these 26 patients, 33/41(80.5%) of microdeletions were in AZFc region. Six out of 41 (14.6%) of microdeletions were in 5F interval between AZFa and AZFb [Figure 1] (in Ys182 primer) and remaining 2/41 (4.9%) of microdeletions were in AZFb region. Five azoospermic cases (2, 9, 12, 22, and 49) and one sever oligozoospermic case (no. 5) had microdeletions in sequences of Ys182 primer [Figure 2], as well KAL-Y gene. This gene (KAL-Y) is classified as a pseudo gene and is inactive homologous of KAL-1 gene on X chromosome [Tables 3a and b].

**Figure 3:**
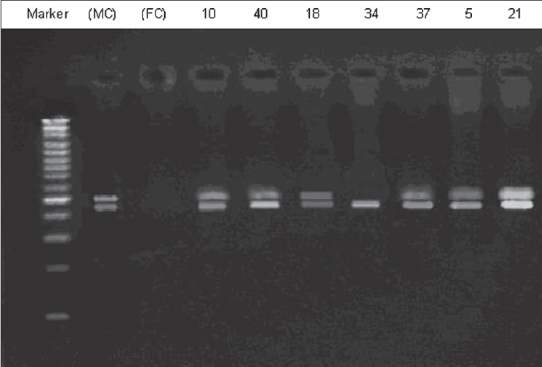
Electroforesis results of multiplex PCR product amplified by sY283 primer in patient 34 with SRY control. Patients’ numbers are indicated at top. Arrows represent the deleted subjects. MC, male control; FC, female control

Multiplex PCR for patient no. 34 with primers, sY283, sY277, and sY153, revealed no band in 497, 312, and 139 bp, but control band was present [Figure 3].

**Figure 4:**
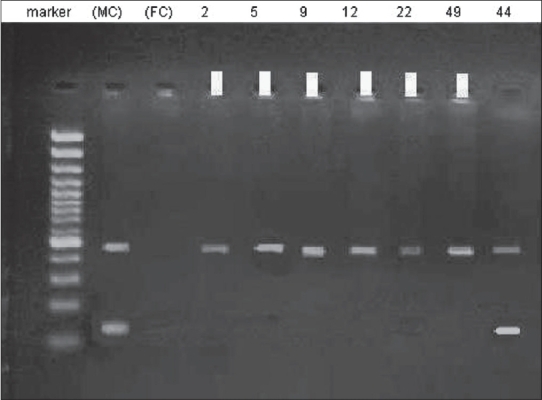
Electroforesis results of multiplex PCR product amplified by sY182 primer in patients’ numbers 2, 5, 9, 12, 22, and 49. Patients’ numbers are indicated at top. Arrows represent the deleted subjects. MC, male control; FC, female control

Multiplex PCR performed for six patients (49, 22, 12, 9, 5, and 2) with SRY and sY182, primer mix. Conform the observed microdeletions in these patients [Figure 4].

In short, 41 microdeletions in 26 azoospermic and sever oligozoospermic patients were present in three deferent regions of AZF gene as following:

*AZFa region* : six patients (nos. 2, 5, 9, 12, 22, and 49) had microdeletions in AZFa region with sY182 primer.

*AZFb region* : two patients (nos. 5 and 40) had microdeletion in AZFb region with sY133 primer.

*AZFc region* : 18 patients also had microdeletions in AZFc region with 14 different primers.

The rapid growth of molecular biology has determined that microdeletions of the Y chromosome represent an important cause of male infertility. These findings are fundamental for a careful diagnosis of male infertility. The detection of a deletion in an infertile man provides a proper diagnosis of the disease and allows the clinician to avoid unnecessary and expensive treatments. It is now clear that a molecular diagnostic test of Y chromosome microdeletions should be at least performed in all azoosperm and oligozoosperm men.

The present study compared data on detection of Y chromosome microdeletions and semen analysis of 50 Iranian males seeking infertility treatment. Y chromosome microdeletions were seen in 16 of 31 males with azoospermia (51.6%) and 10 of 19 with *sever* oligozoospermia (52.6%). The overall frequency of Y chromosome microdeletion detection in the infertile Iranian population in the present study was found to be 26/50 (52%). Although, so far, the incidence of microdeletions in AZF locus in infertile men has not been cleared out, but according to published records in different articles[15] the world frequency of these microdeletions in infertile males is about 5-15% (in azoospermia males 10-15% and in sever oligozoospermia males 5-10%).

Based on comparison research done between infertile males population in France, Denmark and Italy, it have been postulated that the geographical location and under study populations are two effective and important factor in the frequency of microdeletions. These factors can change the frequency, more or less of worldwide statistic standards.[16] The reason for high frequency of our data as compare with other studies could be the small sample size (50 patients) beside factors proposed by Krausz and his colleague.[16]

Najmabadi *et al.*[17] suggests a high prevalence (18%) of Yq microdeletions in men with idiopathic azoospermia/severe oligospermia. The physical locations of these microdeletions provide further support for the concept that a gene(s) on Yq deletion interval 6 plays an important role in spermatogenesis. The presence of deletions that do not overlap with the DAZ region suggests that genes other than the DAZ gene may also be implicated in the pathogenesis of some subsets of male infertility.

Comparison of our data with the result of other investigators worldwide shows that the incidence of Yq microdeletions in this group (South of Iran) is higher than international frequency. In this study, 41 microdeletions in Yq chromosome has been observed in 26 patients. Of 41 microdeletion, 33 (80.5%) are in AZFc region, 6 (14.6%) are in AZFa region, and 2 (4.9%) are in AZFb. The locations of these microdeletions also support the concept that a gene(s) on Yq deletion interval 6 plays an important role in spermatogenesis.[17]

Based on the result of several researches, highest number of AZF locus microdeletions in infertile males, is related to the DAZ/AZFc region. The DAZ (deleted in azoospermia gene region) gene cluster localized on the distal euchromatic region of the Y chromosome (AZFc region) is one of the most important candidate genes involved in infertility. Absence of the DAZ gene cluster is known to cause sterility via meiotic arrest or absence of all germ cells.[18] Kostiner *et al.*[19] have reported that about 6-13% of men with oligozoospermia or azoospermia showed deletions in all or most DAZ gene clusters.

In our study, DAZ genes microdeletion was seen only in one patient and most of the microdeletions (80.5%) occurred in AZFc region and agrees with other studies regarding microdeletions of AZFc, but for microdeletions of AZFa our results shows higher incidence (14.6%) and differ significantly with many studies, although this significance difference remains unexplained, which again could be due to geographical and population differences proposed by Krausz and his colleague.[16]

There will be some problems associated with the use of ICSI in infertile males, as most of the Y chromosome microdeletions may be passed on from father to son and may result in infertility in the son as well. Chang *et al.*[20] reported a family with similar DAZ deletions in a father and his four infertile sons. Kent-First *et al.*[21] demonstrated that deletions in Yq males could be transmitted to their sons when ICSI was performed. They studied sons born through ICSI in 32 infertile fathers and found three affected father/son pairs where microdeletion of different sizes were found in the AZFb and AZFc regions. Therefore, it is essential that the infertile male partners are counseled and semen analysis, karyotyping and Y microdeletion studies, done before ICSI is performed.

## CONCLUSION

The detection of a deletion in an infertile man provides a proper diagnosis of the disease allows the clinician to avoid unnecessary and often expensive treatments to improve fertility and has important ethical consequences if the patient is a candidate for assisted reproduction techniques.

The identification of the actual role played by the *AZF*-candidate genes in spermatogenesis will provide significant advances to our understanding of the biology of spermatogenesis, as well as the analysis of novel Y-chromosomal genes with a potential role in male germ cell development will clarify other important features of this important chromosome.

Azoospermia factor microdeletions are specific for spermatogenic arrest and lead to oligozoospermia or azoospermia. Microdeletion analysis using PCR helps determine the frequency and site of gene deletion and thus the testicular phenotype and also contributes to the determination of an accurate prognosis and ultimately to valuable counseling for couples diagnosed with AZF microdeletions.

There are some potential problems associated with the use of ICSI in infertile males. Most of the Y chromosome microdeletions may be passed on from father to son and may result in infertility in the son as well.
